# Exploring Relationship between Face-to-Face Interaction and Team Performance Using Wearable Sensor Badges

**DOI:** 10.1371/journal.pone.0114681

**Published:** 2014-12-15

**Authors:** Jun-ichiro Watanabe, Nozomu Ishibashi, Kazuo Yano

**Affiliations:** 1 Central Research Laboratory, Hitachi, Ltd., Tokyo, 185-8601 Japan; 2 Hitachi High-Technologies Corporation, Tokyo, 105-8717 Japan; University Toulouse 1 Capitole, France

## Abstract

Quantitative analyses of human-generated data collected in various fields have uncovered many patterns of complex human behaviors. However, thus far the quantitative evaluation of the relationship between the physical behaviors of employees and their performance has been inadequate. Here, we present findings demonstrating the significant relationship between the physical behaviors of employees and their performance via experiments we conducted in inbound call centers while the employees wore sensor badges. There were two main findings. First, we found that face-to-face interaction among telecommunicators and the frequency of their bodily movements caused by the face-to-face interaction had a significant correlation with the entire call center performance, which we measured as “Calls per Hour.” Second, our trial to activate face-to-face interaction on the basis of data collected by the wearable sensor badges the employees wore significantly increased their performance. These results demonstrate quantitatively that human-human interaction in the physical world plays an important role in team performance.

## Introduction

Improving work performance is the main mission of managers in organizations. Studies in psychology and social science have pointed out that group dynamics is a key factor in improving group performance [1]–[4]. A rich trove of human behavioral data has enabled group dynamics to be studied quantitatively [5]. Studies in computational social science have targeted this massive amount of data, which has been collected from sent e-mail logs [6]–[9], mobile phone data [10]–[13], and activity records on social networking services [14]–[16]. Analysis of this data has revealed patterns of individual and group behaviors and has provided great insight into human behavior.

Wearable sensors have been used to measure physical human behaviors in various environments such as the medical field [17]–[19], schools [20]–[23], and various social communities [24], [25] to study human dynamics. Use of these sensors has changed the way human behavior is studied because they can be used to collect quantitative data reflecting face-to-face interaction and bodily movements [26], [27]. Recently, using wearable sensors to measure employee activities in organizations has attracted attention because the data collected can be used for managing the organization and improving productivity [28]. Wearable sensors and devices are used to quantitatively capture employees' activities and provide information without disturbing their work for improving the efficiency of their work processes. Results of recent studies have revealed evidence of dynamics underlying face-to-face interaction patterns among employees and their performance [29]–[31]. However, the quantitative evaluation of the relationship between the two has not been sufficient thus far, and because data have only just been gathered, there is still only a limited amount to work with.

Our objective in this work was to identify the physical behavioral factors that affect work performance. We had employees in call centers wear sensor badges and used the data collected to examine the relationships between their physical behaviors and their performance. The badge-shaped wearable sensor (Figure S1(a) in S1 File [32]) we used is designed to measure physical behaviors by capturing data related to bodily movement and face-to-face interaction. We targeted two inbound call centers, Call Center A and Call Center B, both of which were operated by the same company and handled the same automobile insurance products. Employees who communicate with customers over the phone are generally called telecommunicators (TCs). Supervisors (SVs) walk around the TCs' desks to provide support when needed. We had TCs, SVs, and managers (MGRs) wear the sensor badges to collect their behavioral data from 11th Sept. to 27th Oct., 2013.

## Results

### Measures

We used Calls per Hour (CPH) as a measure to represent call center performance. Here, CPH means the number of phone calls a TC handled in one hour. The larger the CPH, the higher the performance.

**Figure 1:**
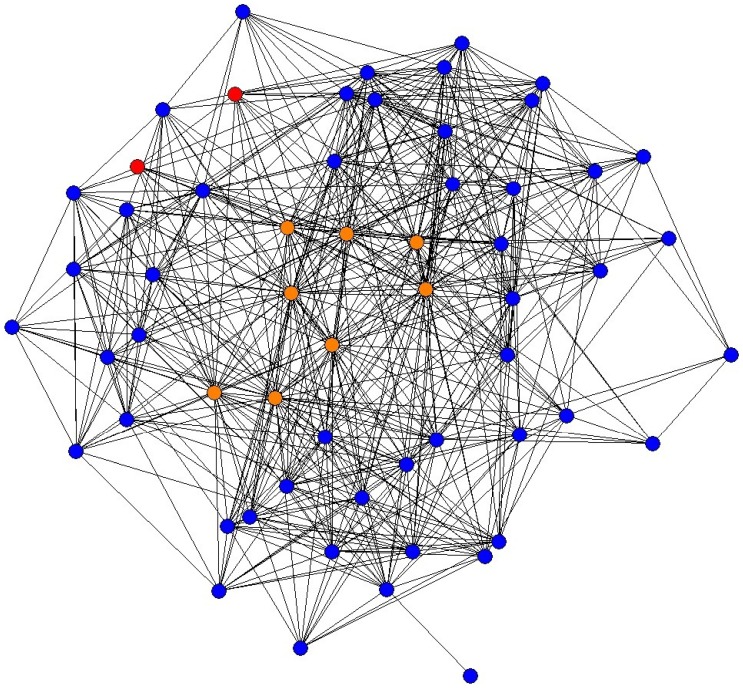
Daily face-to-face network diagram of Call Center A averaged over our experimental period. Telecommunicators (TCs), supervisors (SVs), and managers (MGRs) are represented by blue, orange, and red nodes, respectively. Links are drawn if there was a face-to-face event between two people for a length of time exceeding a predefined threshold (3 min). This diagram is drawn using the spring model, in which nodes with many links are located closer together while those with few links are located further apart. Note that SVs are located at the center of the diagram, indicating that they are “hubs” of face-to-face interaction among employees. TCs located at the edge of the diagram are employees with less face-to-face interaction with others.

The face-to-face interactions were captured using infrared data association (IrDA) transceivers in the badges (S1 File). We consider two people to have interacted with each other if there was a face-to-face event between them exceeding a predefined threshold, which we defined as three minutes per day. Face-to-face interaction among employees can be drawn as a network diagram, an example of which is shown in Fig. 1. Three measures were used to evaluate the cohesiveness or activeness of the mutual face-to-face interaction: degree, clustering coefficient, and geodesic distance [33]. Degree 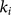 of node 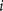 is defined as the number of links connected to the node and represents the number of people an employee met with. The clustering coefficient of node 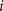 is defined as 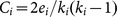, where 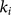 and 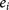 stand for the number of nodes connected to node 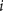 (i.e., degree) and the number of links between them, respectively. This coefficient represents the density of the triangles formed by the three nodes and the links between them. The geodesic distance 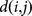 between nodes 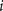 and 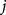 is defined as the minimum number of links between the nodes. We calculated these measures for the entire call center as the average for all employees: 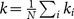, 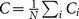, and 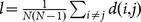, where 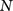 is the total number of employees in the call center. The larger the 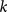 and 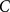, and the shorter the 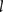, the more cohesive the network structure and the more active the interactions among employees.

The physical movements of the employees were captured using the acceleration sensor in the badges they wore (S1 File). The zero-crossing count, defined as the number of times the acceleration signal crossed the zero-level per unit time, was used to determine the activity level: the higher the count, the more active the person's bodily movements. Each employee's activity level was judged to be in one of two states, active or non-active, every minute in accordance with the zero-crossing count. The threshold frequency for the count was set to 2 Hz on the basis of the results of a preliminary study, which showed that this is the level at which active motion such as conversation with gestures could be distinguished from quieter motion such as keyboard typing. Therefore, the activity level for a minute during which the zero-crossing frequency was greater than 2 Hz was judged to be in the active state and otherwise was judged as non-active. We then defined the activity level of the entire call center as
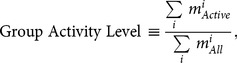
(1)where 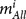 is the total number of minutes employee 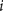 was in a defined period (e.g., the total minutes he or she worked in a day) and 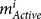 is the total number of minutes he or she was judged to be in the active state during that period. Hereafter, we call the value calculated in eq. (1) the GAL for short. The GAL takes a value of 0 to 1, where a higher value means that the call center was more active.

### Skill Level and Performance

Both call centers evaluate the skill level of its TCs as one of two ranks: 1 (low) or 2 (high). For Call Center A, we observed a significant difference between the average performance of TCs with high skill level and that of TCs with low skill level, as would naturally be expected: the average CPH during our experimental period for TCs with high skill level was 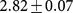 whereas that for TCs with low skill level was 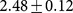 (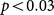). However, for Call Center B, we observed no such significant difference depending on the skill level. Moreover, as shown in Table 1, the average performance of Call Center A during our experimental period was significantly higher than that of Call Center B (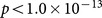), in spite of the fact that there is no significant difference between the average skill level of the TCs (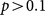). This suggests that performance is determined not only by TC skill level but also by other factors.

**Table 1:** Average performance and skill level of employees.

	Average performance (CPH)	Average skill level of TCs	Participants
**Call Center A**	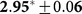	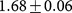	53 TCs, 8 SVs, 2 MGRs
**Call Center B**	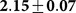	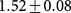	38 TCs, 3 SVs, 2 MGRs

**Figure 2:**
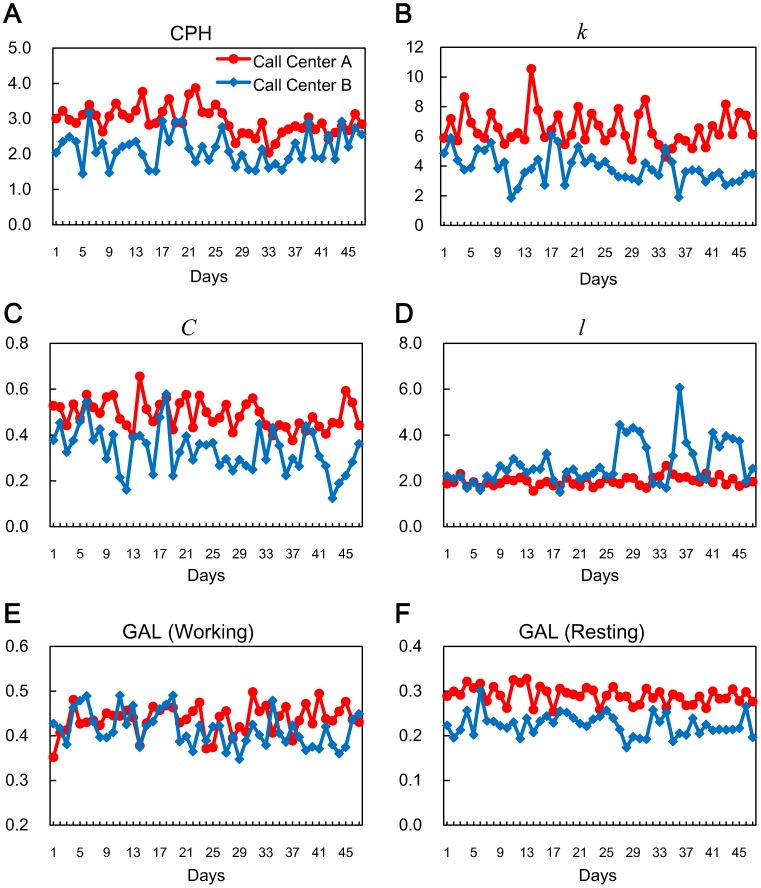
Transition of daily measures averaged over employees working on each day. Data for 47 continuous days (from 11th Sept. to 27th Oct., 2013) are plotted. **A**, CPH. **B**, degree 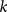. **C**, clustering coefficient 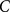. **D**, geodesic distance 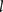. **E**, GAL calculated for time when employees were around their desks, working. **F**, GAL calculated for time when employees were away from their desks, resting. Whether employees were around their desks was determined from data obtained using IR beacons placed on the desks. The daily CPH of both call centers correlated with each other (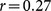, 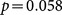, **A**). Call Center A, which had a higher overall performance (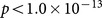, **A**), had a higher 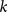 (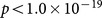, **B**), 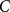 (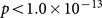, **C**), GAL while working (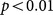, **E**), and GAL while resting (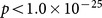, **F**). It also had a shorter 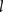 (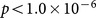, **D**).

Average performance measured as CPH (see also Fig. 2A), average skill level of TCs judged as 1 (low) or 2 (high), and number of participants who wore sensor badges. Values for average CPH and average skill level are mean 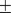 SEM. * indicates a significant difference from Call Center B (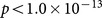).

### SV-TC Interaction and Performance

In both call centers, SVs walk around the TCs' desks to give them support if needed and to encourage them to elicit better performance. This random intervention is assumed to be effective for improving their performance at the job scene. We evaluated the effect of this random intervention on their performance using data obtained by the wearable sensor badges.

To see if a TC who is well taken care of by SVs gets a higher performance, we calculated the daily average time a TC faced with SVs to examine the correlation with individual CPH averaged over the experimental period. However, we observed no significant correlation between the two (Pearson's correlation coefficient: 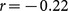, 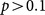 for TCs in Call Center A and 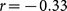, 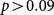 for TCs in Call Center B). This means that a TC who was taken care of by SVs longer did not necessarily get a higher performance.

Next, we examined the relationship between the degree of intervention and the performance of the entire call center. We calculated the daily average time an SV faced any TCs and examined the correlation with the daily CPH of the entire call center (i.e., value averaged over TCs who worked on a particular day). Again, we observed no significant correlation between the two (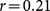, 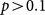 for Call Center A and 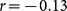, 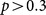 for Call Center B). We also calculated the average number of TCs an SV faced each day but observed no significant correlation with the daily CPH of the call center (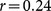 for Call Center A and 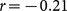 for Call Center B; both 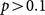). These results indicate that intervention time or the number of TCs an SV interacted with, which can be regarded as measures representing the presence of SVs, does not necessarily directly affect the call center performance. In other words, the effect of random intervention is not guaranteed, contrary to the commonly held belief on the job scene.

### Individual Behaviors and Performance

We then focused on not only SV-TC interaction but also the face-to-face interaction structure among all employees and the bodily movement of them.

We calculated daily degree 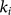, clustering coefficient 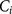, geodesic distance 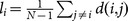, activity level 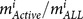, and CPH averaged over our experimental period for each TC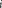 to examine the relationship between individual physical behaviors and his or her performance. We found no significant correlation between these physical behavioral measures and individual performance (Table 2). This indicates that how an individual behaves does not relate to individual performance: i.e., a TC who faced others more or who was always active did not necessarily get a higher performance. This is in line with the previous studies [21], [31].

**Table 2:** Correlation coefficients between individual CPH and behavioral measures.

	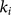	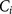	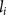	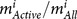
**Call Center A**	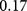	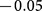	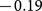	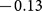
**Call Center B**	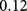	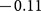	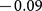	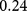

Pearson's correlation coefficients between individual physical behavioral measures and CPH are calculated. No significant correlation was observed.

### Face-to-Face Interactions and Team Performance

Next, we examined the relationship between collective physical behaviors and the team performance, that is, the CPH of the entire call center averaged over the employees. We compared the daily physical behaviors at the two call centers. Fig. 2B, C, and D shows transitions of daily 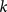, 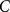, and 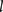 for both call centers. The face-to-face interaction measures of Call Center A, which had quite a high performance (Fig. 2A, Table 1), were significantly higher (for 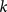 and 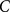) or shorter (for 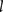) than those of Call Center B (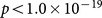 for 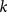; 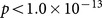 for 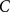; 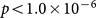 for 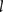). Similarly, we found that the daily GALs of Call Center A were significantly higher than those of Call Center B (Fig. 2E and F). Especially significant was the difference of GALs calculated only for resting time (Fig. 2F, 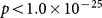). Here, we distinguished working time from resting time by using IR beacons placed on the employees' desks (see Figure S1(b) in S1 File). We define resting time as any time when employees are not in the working room, which is where TCs basically communicate with customers by phone from their desks and SVs and MGRs walk around the desks providing any needed support. Whether they were physically in the working room or not was determined from the data collected by the IR beacons on the TCs' desks. If there was no face-to-face event between a sensor badge and any IR beacons on the desks, the employee wearing that badge was assumed to be not in the working room but out on a break. Employees spent their break times in the break room, restroom, or smoking area. Though the difference of GALs calculated for working time were also significant (Fig. 2E, 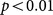), the difference in GAL while resting was more remarkable.

We further investigated the relationship between the physical behaviors of employees and the daily performance at each call center. Table 3 shows correlation coefficients between daily physical behavioral measures and daily performance. For Call Center A, we observed a significant correlation between daily performance and face-to-face interaction among employees represented in clustering coefficient 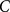 (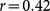, 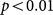) and geodesic distance 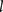 (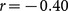, 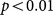), and a weak correlation for degree 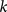 (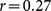, 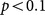). For Call Center B, we observed a weak correlation between daily performance and face-to-face interaction represented in geodesic distance 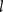 (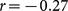, 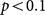). Also for Call Center B, we observed a significant correlation between GAL while resting and performance (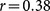, 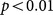).

**Table 3:** Correlation coefficients between daily CPH of call center and behavioral measures.

	Behavioral measures	Correlation coefficient
**Call Center A**	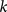	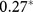
	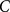	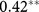
	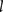	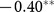
**Call Center B**	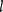	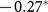
	GAL while resting	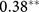

Pearson's correlation coefficients are calculated. * and ** indicate 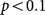 and 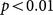, respectively.

The results concerning GAL (Fig. 2E, F, and Table 3) raise a question: What causes the higher GAL? We found that GAL is determined mainly by face-to-face interaction. GAL calculated using data when two or more people were physically facing each other was significantly larger than that calculated using data when a person was not facing anyone. Specifically, the average daily GAL while facing was 0.38 whereas that while not facing was 0.26 (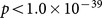). This could be caused by bodily movements accompanying conversation with gestures.

### Causality

These results (Fig. 2, Table 3) indicate that there is a significant relationship between face-to-face interaction (or GAL caused by the face-to-face interaction) and team performance. Specifically, a call center or days with more active face-to-face interaction seems to result in higher performances. However, we need to clarify not only the correlation but also the causality to see if the face-to-face interaction is the controllable determinant for managers to improve performance. Essentially, does face-to-face interaction affect performance or does performance affect face-to-face interaction?

To clarify this causality, we conducted a two-week trial at Call Center A and changed a key detail of the face-to-face interaction among employees by having SVs talk to TCs who had less face-to-face interaction with others (i.e., lower 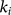) based on the data collected by the sensor badges, thus activating face-to-face interaction. The trial was conducted from 28th Oct. to 10th Nov., 2013, immediately after completing our initially planned experimental period on 27th Oct. SVs were informed of the objective of this trial in advance—specifically, that the intent was to improve performance by changing face-to-face interaction among employees—while TCs were not informed of this objective and were told simply that the experimental period for measuring the face-to-face interaction among employees was extended. As a control condition, the employees at Call Center B continued to wear the sensor badges with the usual call center administration, in which random intervention by SVs were conducted, during the same period.

**Figure 3:**
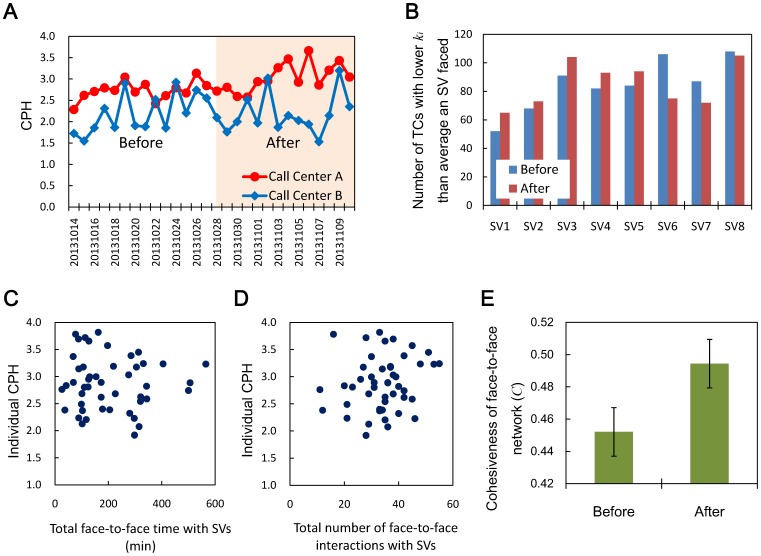
Results of trial to improve face-to-face interaction among employees. **A**, transition of daily CPH. **B**, number of TCs who had a lower 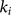 than average before starting the trial each SV faced. **C**, scatter plot of individual daily CPH averaged over the trial period and total face-to-face time with SVs during the trial period. **D**, scatter plot of individual daily CPH averaged over the trial period and total number of interactions with SVs during the trial period. **E**, change of clustering coefficients 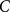 of face-to-face network. “Before” is the period from 14th to 27th Oct. “After” is the period from 28th Oct. to 10th Nov., during which the trial was conducted. The average CPH of Call Center A significantly increased (from 2.73 to 3.03, 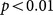, **A**) whereas that of Call Center B did not. During the trial, 5 of 8 SVs (SV1–SV5) at Call Center A faced more TCs who had fewer interactions with others (i.e., TCs with lower 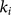 than the average) before starting the trial (**B**). There were no significant correlations between face-to-face time or number of face-to-face interactions with SVs and individual performance (**C** and **D**). Cohesiveness of face-to-face interaction reflected in 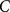 increased significantly (from 0.45 to 0.49, 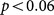, **E**). Error bars refer to SEMs.

We compared the daily performance of both call centers for the trial period and the same length of period before starting the trial (from 14th Oct. to 27th Oct). We observed a significant increase in the daily CPH of Call Center A (Fig. 3A: 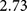 on average for the two weeks before starting the trial and 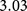 on average during the trial, 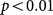) whereas we observed no such significant change in the CPH for Call Center B (from 2.19 to 2.18 on average, 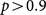). This indicates that the trial did work.

We found that during the trial period, 5 of 8 SVs at Call Center A faced more TCs who had fewer interactions with others (i.e., TCs with a lower 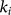 than the average) before starting the trial (Fig. 3B). However, the daily averaged time an SV faced TCs showed no significant increase (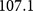 to 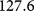 min, 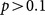). This indicates that SVs tried to take care of the TCs with a lower 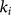 following the trial guidance in their limited working hours. We found no significant correlation between face-to-face time or number of SV-TC interactions and individual performance during the trial period (Fig. 3C, D). This means that, as discussed in the previous section, intervention itself does not directly affect individual performance.

Then, how did the trial work? The results from the first experiments (Fig. 2, Table 3) suggest that during the trial, the face-to-face interaction structure among all employees must have changed, and that this change affected the performance. Actually, we observed a significant increase in the cohesiveness of face-to-face interaction among employees represented as clustering coefficient 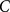 averaged over the trial period compared to that averaged over the same time period before starting the trial (Fig. 3E, from 0.45 to 0.49, 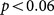). As we can see in Fig. 1, SVs function as hubs in the face-to-face interaction. In the trial, having SVs interact with TCs who had less interaction with others essentially stretched the links between hub nodes and nodes with fewer links located at the edge of the face-to-face network. This increased the triangle structure, that is, the cohesiveness of the face-to-face interaction among employees. Considering the correlation between face-to-face interaction measures and CPH (Table 3), this result clarifies the causality: essentially, the increase of cohesiveness of face-to-face interaction caused by the trial improved the performance.

## Discussion

We have shown that face-to-face interaction among employees significantly correlates with the call center's performance. We need to consider other factors that might affect call center performance. Although there is a significant difference between the performances of both call centers (Fig. 2A), we found that the daily CPH of both call centers correlated with each other (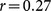, 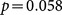). This indicates that the CPH is definitely affected by possible external factors, such as economical trend, or by seasonal factors. In our trial, we changed the face-to-face interaction structure in Call Center A and did nothing for Call Center B. The results of this trial (showing that the CPH of Call Center A significantly increased whereas that of Call Center B did not) indicate that not only external factors but also face-to-face interaction could affect the team performance.

Here, we should mention the effect of intervention in the trial in which SVs were asked to talk to TCs with lower face-to-face interaction on the basis of data collected by sensor badges. As we discussed in the previous sections, our analysis showed that the intervention itself, represented as face-to-face time between SV and TC or frequency of SV-TC interaction, does not directly affect the individual performance (see Fig. 3C, D). This indicates that the possible increase of the presence of SVs caused by the trial did not directly affect the performance increase. We found that the structure of face-to-face interaction among employees or activeness of workplace (GAL) caused by the face-to-face interaction is what affects team performance. We think that targeting TCs with a lower degree of face-to-face interaction with others for the intervention worked effectively and certainly to increase the cohesiveness of face-to-face interaction among employees.

We have seen that GAL is related to face-to-face interaction: GAL while facing another person is significantly higher than that while being alone. Therefore, the difference of GALs between the two call centers (Fig. 2E and F) must be caused by differences of face-to-face patterns. Table 4 shows the average intervals and durations of face-to-face events calculated while working and while resting for the two call centers. As for the intervals between the face-to-face events while resting, we observed a significant difference between the two call centers (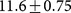 min for Call Center A vs. 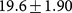 min for Call Center B, 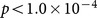). The employees at Call Center B took about double the number of minutes when encountering colleagues during breaks compared to employees at Call Center A. This difference of intervals could explain the significant difference of GAL while resting (Fig. 2F). As for durations of facing others while working, we observed a significant difference (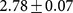 min for Call Center A vs. 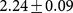 min for Call Center B, 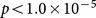): on average, the employees at Call Center A continued facing others more than 30 seconds longer than employees at Call Center B. This difference could be what causes the significant difference of GAL while working (Fig. 2E). This result suggests that not only the spatial cohesiveness of a face-to-face network represented as 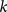, 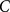, or 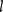 but also the temporal patterns of encountering colleagues might relate to performance.

**Table 4:** Temporal face-to-face patterns.

	Intervals [min]		Duration [min]	
	Working	Resting	Working	Resting
**Call Center A**	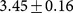	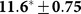	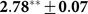	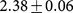
**Call Center B**	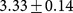	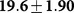	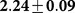	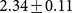

Interval time between face-to-face events and duration of facing time calculated separately for working and resting time. Values are mean 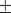 SEM. *and ** indicate 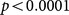 and 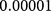 from Call Center B, respectively.

We should also consider the effect of team composition on the face-to-face interaction and team performance. The number of employees was not same between the two call centers (Table 1). This could explain the differences we found in the measures (Fig. 2). Another issue we should consider is the differences between the break rooms of the two call centers. In Call Center A they have large break room that enables many employees to come together, whereas in Call Center B they have a much smaller one. This difference of break rooms could affect the way employees spend their break times and account for the difference of GAL while resting (Fig. 2F). We feel that our findings indicate a possible relationship between designing a team or workplace environment and the cohesiveness of face-to-face interaction among employees, which in turn has a positive impact on employee performance.

Our study has some obvious limitations. It was conducted in a particular country, and it targeted inbound call centers. A study conducted in a country with different social and cultural backgrounds or for a different business field might produce different results. We think that it is important to consider these social and cultural diversities to obtain more reliable results. Our results indicating the relationship between physical behaviors while resting and performance are consistent with results of studies focused on different countries [29] or fields [21], [31]. Although we still need to gather more data from more call centers and in more varied fields in order to identify the universality, we feel our findings here have revealed a fundamental mechanism underlying collective human behaviors in informal situations and team performance. Though the correlations found in the two call centers are significant (Table 3), we think that we need to conduct additional experiments at other call centers to see if there are any common measures which affect call center performance. We showed that intervention by SVs itself did not increase the TCs' performance directly (Fig. 3C, D). However, we think that there still remains a possibility of any special reason, such as the Hawthorne effect, for the performance increase. We plan to design a trial in which the structure of face-to-face interaction will be changed without considering the effect of the intervention to examine the relationship between face-to-face interaction and performance more effectively.

In summary, we used wearable sensors to explore the physical behavioral factors of employees as they relate to work performance. Analyses of data from two call centers revealed that face-to-face interaction among employees is significantly correlated with performance. Furthermore, the results of a trial encouraging the activation of face-to-face interaction demonstrated that face-to-face interaction could be a determinant of team performance. These results demonstrate quantitatively that physical collective behaviors play an important role in team performance.

## Materials and Methods

### Dataset

Physical behavioral data were obtained by having employees of two call centers wear sensor badges from 11th Sept. to 10th Nov. 2013 (61 continuous days). Participants at Call Center A included 53 TCs (46 females, 7 males), 8 SVs (2 females, 6 males), and 2 managers (males) and those at Call Center B included 38 TCs (26 females, 12 males), 3 SVs (females), and 2 managers (1 male, 1 female). Participants put on the sensor badges every morning when they arrived at the office and returned them to a charging cradle when they went home for the day. Primal analyses were done automatically by a server-side application to calculate a daily adjacency matrix representing face-to-face events among subjects and zero-crossing counts of acceleration signals for each subject every minute. Face-to-face interaction measures or GAL were calculated based on these primal data. The daily calculated measures are available to the public as Dataset S1 and Dataset S2 in S2 File. Personal information including age, gender, skill level, and position were obtained from the call centers before starting the experiment. Daily work data including the number of calls and log-in time to Private Branch Exchange (PBX) were also provided by the call centers to enable us to calculate the CPH.

### Ethical Statement

Written informed consent was obtained from all participants after they were informed of the purpose, procedures, risks, benefits, and voluntary nature of the experiments. This study was approved by the internal review board of the Central Research Laboratory, Hitachi, Ltd., and personal information and behavioral data were obtained according to the regulations of this review board. The collected data were provided only to Hitachi High-Technologies Corp. and Hitachi CRL, and not to any third parties.

### Statistical Analysis

The Student's *t*-test was used to compare the mean parameter values in Tables 1 and 4. Pearson's correlation coefficients were calculated for evaluating the correlations in Tables 2 and 3.

## Supporting Information

S1 File **Supplementary text and figures.** Figure S1(a), Wearable sensor badge. Figure S1(b), IR beacon.
(PDF)

S2 File **Dataset S1 and Dataset S2.** Dataset S1, Daily measures of Call Center A. Dataset S2, Daily measures of Call Center B.
(ZIP)
