# Supplementary material for: Exploring Relationship between Face-to-Face Interaction and Team Performance Using Wearable Sensor Badges
Source: PLoS One. 2014 Dec 15;9(12):e114681. doi: 10.1371/journal.pone.0114681 (PMC4266505; doi:10.1371/journal.pone.0114681)
Supplement: S1 File — Supplementary text and figures. Figure S1(a), Wearable sensor badge. Figure S1(b), IR beacon. (PDF) [file pone.0114681.s001.pdf]

Supporting Information

Exploring Relationship between Face-to-Face Interaction and Team Performance Using Wearable Sensor Badges

by Jun-ichiro Watanabe, Nozomu Ishibashi, and Kazuo Yano

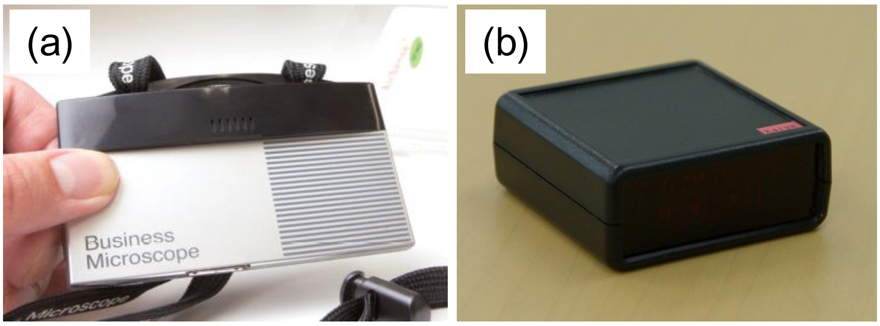

Figure S1. (a) Wearable sensor badge and (b) IR beacon.

The badge-shaped wearable sensor (Fig. S1 (a)) we used is designed for measuring physical behaviors. It captures data related to bodily movement and face-to-face interaction. The badge is light and small enough to be hung from a person's neck during working hours.

Data on the wearer's physical movements are captured by a three-axis micro electro mechanical system (MEMS) acceleration sensor and are used to detect individual activities such as being quiet, keyboard typing, conversation with gestures, and walking. Six infrared data association (IrDA) transceivers on the front of the badge and facing different angles are used to detect face-to-face events. They can transmit and receive signals up to a distance of 3 m within a 15° cone. Two face 15° leftward horizontally, two face 15° rightward horizontally, and the other two face forward and 30° downward. With this alignment, the overall detection range is 60° horizontally and vertically, which should cover virtually any face-to-face interaction in a workplace environment. The transceivers are sequentially powered on and off to reduce power consumption. By using user IDs linked to each badge, information can be obtained about who met whom, when, and for how long.

Location information is obtained by using IR beacons (Fig. S1(b), 65 × 65 × 28 mm) set at particular places such as the break room and the smoking area. Four IrDA transceivers on the front of the beacon, facing slightly different angles, create a detection range that covers 60° horizontally and 30° vertically. A face-to-face event between badge-wearing individuals is detected on the basis of interactions between the transceivers in their badges and an IR beacon. This means that, in addition to getting information about who met whom, when, and for how long, we also obtain location information.

In the experiment, we placed the IR beacons on the employees' desks to distinguish working time from resting time. We define resting time as any time when employees are not in the working room, which is where TCs basically communicate with customers by phone from their desks and SVs and managers walk around the desks providing any needed support. Whether they were physically in the working room or not was determined from the data collected by the IR beacons on the desks. If there was no face-to-face event between a sensor badge and any IR beacons on the desks, the employee wearing that badge was assumed to be not in the working room but out on a break.

The captured data are stored in built-in 32-MB flash memory and offloaded for database transfer while the badge is in the charging cradle overnight. The badge is roughly the same size as a credit card: it is 86 × 54 × 7 mm and weighs 34 g, including the battery. The battery lasts about 24 hours between charges.
